# Enzyme-Treated Soybean Meal Replacing Extruded Full-Fat Soybean Affects Nitrogen Digestibility, Cecal Fermentation Characteristics and Bacterial Community of Newly Weaned Piglets

**DOI:** 10.3389/fvets.2021.639039

**Published:** 2021-05-20

**Authors:** Hao Li, Jie Yin, Xi He, Zhiqing Li, Bie Tan, Qian Jiang, Jiashun Chen, Xiaokang Ma

**Affiliations:** College of Animal Science and Technology, Hunan Agricultural University, Changsha, China

**Keywords:** bacterial community, hindgut fermentation, dietary protein, gut health, weaned piglets

## Abstract

The study investigated the impact of soybean protein from different processing on the performance, dietary nitrogen digestibility, cecal fermentation characteristics, and bacterial community in newly weaned piglets. The piglets were allocated to two dietary treatment and fed with the extruded full-fat soybean diet (EFS group) and enzyme-treated soybean meal diet (ESBM group), respectively. The piglets in ESBM group showed greater nitrogen digestibility and feed efficiency, and lower diarrhea rate in comparison to piglets in EFS group (*P* < 0.05). Cecal samples from piglets in ESBM group contained greater concentration of acetate, propionate and total SCFAs (*P* < 0.05), and lower contents of isobutyrate, isovalerate, total BCFAs, NH_3_-N and putrescine (*P* < 0.05) than cecal samples from piglets in the EFS group. The cecal samples from piglets in ESBM group contained greater abundances of g_*Blautia*, g_*Coprococcus_3*, g_*Fusicatenibacter*, and g_*Bifidobacterium* than the cecal sample from piglets in the EFS group, which could promote to protect intestinal health. In summary, enzyme-treated soybean meal may enhance the growth performance of weaned piglets *via* increasing the dietary nitrogen digestibility, preventing protein fermentation in the hindgut, which shed light on the mechanism in regulating gut health of dietary protein.

## Introduction

In the pig industry, piglets often suffer from weaning stress, including symptoms such as diarrhea, anorexia, and growth retardation due to immature digestive tract and the change of diets. The reduction of protein fermentation in the large intestine alleviates weaning stress and prevents diarrhea, which is usually achieved by feeding low-protein diets and improving the ileal digestibility of protein (1–3). Soybean is the most widely used protein source in the diets of pigs, but it contains soybean antigens and many other anti-nutritional factors, such as glycinin, β-conglycinin, and trypsin inhibitor, that caused allergic reactions or inhibited the activity of digestive enzymes, thus reducing nutrient digestibility and growth performance of livestock (4, 5). Therefore, improving the utilization of soybean protein in a low-protein diet could have a positive impact on the growth and health for the newly weaned piglets.

Extruded full-fat soybean (EFS) is the soybean treated with extrusion and a common soybean protein source, containing lower toxin, urease, and trypsin inhibitor activity, therefore can improve nutrient digestion and utilization (6). EFS in the diet has been reported to improve growth performance of weaned piglets by increasing the apparent total tract digestibility of crude protein and organic matter, and reducing the rate of diarrhea (7). Enzyme-treated soybean meal (ESBM) is a new type of protein source in piglet diets, which is hydrolyzed from soybean meal by biological enzymes. Compared with soybean meal, ESBM contains higher crude protein, amino acid and small peptide content, and lower anti-nutritional factor content, therefore it has great potential to improve the gut health of weaned piglets (8). Correspondingly, our previous studies have shown that ESBM improved the growth performance, antioxidant index and immune function of weaned piglets (9).

Given that there are only few studies aiming to evaluate the nutritional value of different processed soybean protein in newly weaned piglets, the current study aimed to compare the effects of dietary ESBM and EFS on growth performance, nutrient digestibility, cecal microbiota, and its fermentation characteristics based on a low-protein and AA supplemented diets.

## Methods and Materials

The animal handling and all procedures of this study received approval from the Animal Care and Use Ethics Committee of the Hunan Agricultural University (Changsha).

The ESBM used in this experiment was provided by Hamlet Protein Company (Horsens, Denmark). The EFS used in this experiment was provided by Hunan Lifeng Biological Technology Co., Ltd. (Changsha, China). Table 1 shows the anti-nutrient factors and nutrient composition of EFS and ESBM.

**Table 1:** Anti-nutrient factors and nutrient composition in extruded and enzyme-treated soybean meal (%, as-fed basis)^*^.

**Items**	**EFS**	**ESBM**
**Anti-nutrient factors (ANF)**		
Glycinin, mg/g	10.40	0.32
β-conglycinin, mg/g	7.90	0.15
Trypsin inhibitor, TIU/mg	0.82	0.73
**Nutrient composition, %**		
Dry matter	89.68	93.49
Crude protein	35.53	53.08
Ether extract	16.05	2.23
Neutral detergent fiber	18.20	15.92
Acid detergent fiber	9.36	6.51
Ash	6.54	7.59
Gross energy, MJ/kg	20.83	18.82

* *Data are the means of two replicates of analyzed values*.

*EFS, extruded full-fat soybean; ESBM, enzyme-treated soybean meal*.

### Animals and Dietary Treatments

Thirty-two Duorc × (Landrace × Yorkshire) growing barrows were allotted to 2 dietary treatments in a completely randomized design with 16 repetitions per treatment according to their body weight (BW, 7.91 ± 0.58 kg). The dietary treatments include EFS diet and ESBM diet: the EFS diet was formulated by adding 24% EFS, while the ESBM diet was formulated by adding 12% EFS and 7% ESBM, and corn and soybean oil was increased to balance the energy and protein levels (Table 2). The experimental diets and vitamin-mineral premix were configured to meet the nutritional needs of nursery piglet as recommended by the NRC (10). Chromic oxide was included in all diets at 0.25% as an indigestible marker in the two diets. The experimental period lasted for 7 days. The house, feed trough and drinker were thoroughly cleaned and disinfected before starting the experiment. The temperature of the pig house was kept at 24–28°C, and the relative humidity was controlled at 60–70%. All the pigs were provided *ad libitum* access to water and feed. A scoring system was applied to indicate the presence and severity of diarrhea as following: 1 = hard feces; 2 = slightly soft feces; 3 = soft, partially formed feces; 4 = loose, semiliquid feces; and 5 =watery, mucous-like feces.

**Table 2:** Ingredients composition and nutrient levels of the experimental diets (%, as-fed basis).

**Ingredients**	**EFS**	**ESBM**
Corn	32.43	36.79
Wheat	20.00	20.00
Enzyme-treated soybean meal	0.00	7.00
Extruded full-fat soybean	24.00	12.00
Whey powder	12.00	12.00
Fish meal	5.00	5.00
Soy oil	3.01	3.63
Dicalcium phosphate	1.05	0.95
Limestone	0.48	0.60
Salt	0.30	0.30
Lysine	0.52	0.55
Methionine	0.09	0.10
Threonine	0.18	0.18
Tryptophan	0.04	0.04
Valine	0.15	0.11
Chromic oxide	0.25	0.25
Vitamin-mineral premix,^a^ no antibiotic	0.50	0.50
**Calculated nutrient levels**		
Metabolized energy, kcal/kg	3,350	3,350
Crude protein	18.52	18.51
Standardized ileal digestible lysine	1.35	1.35
Standardized ileal digestible methionine	0.39	0.39
Standardized ileal digestible threonine	0.79	0.79
Standardized ileal digestible tryptophan	0.22	0.22
Standardized ileal digestible valine	0.86	0.86
**Analyzed nutrient levels**		
Gross energy, MJ/kg	17.46	17.45
Ether extract	5.96	5.40
Neutral detergent fiber	16.50	15.50
Acid detergent fiber	4.10	3.90
Dry matter	90.10	89.90
Ash	6.30	5.70

a *The components and contents of the premix providing nutrients for per kg feed are as follows: Vitamin A, 12,000 IU; Vitamin D3, 2,500 IU; Vitamin E, 30 IU; Vitamin K3, 30 mg; Vitamin B12, 12 micrograms; Riboflavin, 4 mg; Pantothenic acid, 15 mg; Niacin, 40 mg; Choline chloride, 400 mg; Folic acid, 0.7 mg; Vitamin B1, 1.5 mg; Vitamin B6, 3 mg; Biotin, 0.1 mg; Manganese, 40 mg; Iron, 90 mg; Zinc, 100 mg; Copper, 8.8 mg; Iodine, 0.35 mg; Selenium, 0.3 mg*.

*EFS, extruded full-fat soybean; ESBM, enzyme-treated soybean meal*.

### Sample Collection and Processing

The daily feed intake and body weight (BW) of each pig were recorded on the d 0 and d 7 to calculate the average daily gain (ADG), average daily feed intake (ADFI) and feed conversion rate (ADG: ADFI, G: F). Six piglets per treatment were humanely killed by intramuscular injection of serazine hydrochloride (Jilin Huamu Animal Health Product Co., Ltd., Changchun, China) at a dosage of 0.5 mL/kg BW on d 7. Pigs were dissected along the belly line, then the ileum and cecum segments were isolated to collect the digesta samples. Cecal digesta samples were stored at −80°C for microbiome and metabolite analysis and ileal digesta samples were stored at −20°C for subsequent chemical composition analysis to calculate nutrient digestibility.

The feed samples and ileal digesta after freeze-drying were weighed in parallel samples for analysis and determination. Dry matter (DM, AOAC 930.15) and CP (AOAC 984.13) contents were determined following the AOAC (11) procedures. The amino acid (AA) profiles were detected by HPLC (Agilent 1200, Agilent Technologies, USA). Lysine and threonine were detected after hydrolyzing with 6 mol/L HCl at 105°C for 24 h. Methionine was analyzed as methionine sulfone after cold performic acid oxidation overnight before hydrolysis. Tryptophan was determined after hydrolyzing with 4 mol/L LiOH at 110°C for 20 h. The apparent ileal digestibility (AID) of amino acids and nitrogen were calculated using the following formula:

(1) $$\begin{array}{l} \text{AID of diet component}=[(\text{Diet component/Chromium})\text{ d}\\ \;-(\text{Diet component/Chromium})\text{ i}]\\ \;*1/(Diet\;component/Chromium)\;d. \end{array}$$

Where (Diet component/Chromium) d= ratio of diet component to Chromium in the diet and (Diet component/ Chromium) i = ratio of diet component to Chromium in the ileal digesta (12).

### Analysis for Bacterial Microbiota by 16S rDNA

Total genomic DNA of 10 digesta samples were extracted using a Stool DNA Isolation Kit (Tiangen Biotech Co., Ltd., Beijing, China) following the manufacturer's instructions. The quantity and quality of extracted DNAs were measured using a NanoDrop ND-1000 spectrophotometer (Thermo Fisher Scientific, USA) and agarose gel electrophoresis, respectively. One sample from each treatment group was abandoned after DNA extraction failed. The genes of bacteria 16S ribosomal RNA in the region of V4–V5 were amplified by using polymerase chain reaction (PCR) with primers (515F 5′-barcode- GTGCCAGCMGCCGCGG-3′) and (907R 5′-120CCGTCAATTCMTTTRAGTTT-3′). Electrophoresis was applied to analyze the integrity of PCR amplicons by using a Tapestation Instruction (Agilent technologies, USA). AxyPrep DNA Gel 122Extraction Kit was chosen to extract and purify PCR amplicons using 2% agarose gels (Axygen 123Biosciences, Union City, CA, USA) and then the production was quantified using QuantiFluor™ -ST and sequenced on an Illumina MiSeq system. QIIME software was used to demultiplex and quality-filtered raw Illumina fastq files. Operational taxonomic units (OTUs) were defined as a similarity threshold of 0.97 using UPARSE. Then UCHIME was applied to identify and delete the unnormal gene sequences. RDP database (http://rdp.cme.msu.edu/) was also referenced to take the taxonomy-based analysis for OTUs using RDP classifier at a 90% confidence level. The original contributions presented in the study are publicly available. This data can be found here: NCBI SRA database (sequence number: PRJNA720093).

### Phylogenetic Investigation of Communities by Reconstruction of Unobserved States (pICRUSt) Analysis for Predicted Metabolic Functions of Microbial Communities

The result of 16S rRNA gene sequencing was applied by PICRUSt analysis to predict metabolic functions of the bacterial community in the cecal samples of piglets. The resultant OTU table was then used to predict metabolic functions by referencing the Kyoto Encyclopedia of Genes and Genome (KEGG) orthology (KO) database.

### Analysis of Cecal Fermentation Characteristics

The concentration of volatile fatty acids (VFAs) including short chain fatty acids (SCFAs) and branched chain fatty acids (BCFAs) in digesta were analyzed using a gas chromatographic method. Briefly, ~1.0 g of feces were first homogenized in the 1.5 mL deionized water. After centrifuged at 15,000 × *g* at 4 °C for 10 min, supernatants (1 mL of each) were acidified with 25% metaphosphoric acid at a 1: 5 ratio (1 volume of acid for 5 volumes of sample) for 30 min on ice. The sample was injected into a GC 2010 series gas chromatograph (Shimadzu, Japan) equipped with a CP-Wax 52 CB column 30.0 m × 0.53 mm i.d (Chrompack, Netherlands). The injector and detector temperatures were 75 and 280°C, respectively. All procedures were performed in triplicate and total VFAs were determined as the sum of analyzed SCFAs (acetate, propionate, butyrate, and valerate) and BCFAs (isobutyrate and isovalerate).

The fresh cecal digesta samples were collected on d 7 for the analysis of the NH_3_-N and biogenic amine contents, including Putrescine (PUT), cadaverin (CAD), spermine (SPD), and spermidine (SPM). The NH_3_-N was measured by colorimetry according to the manufacturer's instructions (Nanjing Jiancheng Bioengineering Institute, Jiangsu, China). The biogenic amine was measured using HPLC as the main instrument. Weigh about 0.5 g of samples, add pre-cooled 5% perchloric acid, and homogenize for 300 s. Then ultrasonic extraction in a low temperature water bath for 1 h. Centrifuge at 5,000 r/min for 10 min, remove 0.5 ml of supernatant to a 15 ml centrifuge tube. Then, add 1 mL NaOH solution and 20 μl benzoyl chloride, vortex for 30 s in a liquid mixer, place in a 37°Cwater bath for 20 min, and vortex for 30 s every 5 min. After the derivatization is completed, add 2 ml saturated sodium chloride solution, 2 ml anhydrous ether, shake and mix, pipette the supernatant liquid, use nitrogen to blow it to dryness, add 1 ml methanol (HPLC grade) to dissolve, and pass the 0.22 μm filter membrane before the measurement. The test conditions were as following: wavelength: 254 nm; flow rate: 1 ml/min; column temperature: elution at 40°C; Acetonitrile: 0.02 mol/L; Ammonium formate = 30:70 (V: V).

### Statistical Analysis

All data were analyzed by the GLM procedure of SPSS 21.0 (SPSS Inc., Chicago, IL, USA), and each piglet was regarded as a statistical unit. Data are showed as Mean values + standard error of the total mean (SEM). For all tests, *P* < 0.05 was considered as significant difference, while 0.05 < *P* <0.10 as a tendency.

## Results

### Growth Performance

No differences were observed in final BW and ADFI between the two dietary treatments (Table 3). Compared to the EFS diet, ESBM increased the G/F (*P* < 0.05) and tended to improve the ADG of piglets (0.05 < *P* < 0.10). The ESBM diet also decreased the diarrhea incidence throughout the trial period compared to EFS (Table 3).

**Table 3:** Effects of protein source on performance of weaned pigs.

**Items**	**EFS**	**ESBM**	**SEM**	***P*-value**
Initial BW, kg	7.90	7.92	0.07	0.91
Final BW, kg	9.83	10.01	0.12	0.32
ADG, g	275.51	298.98	7.75	0.08
ADFI, g	394.12	403.57	14.64	0.66
G: F	0.68	0.75	0.01	0.01
Diarrhea incidence, %	5.95	1.93	0.57	< 0.01

*Data were shown as the mean + SEM (n = 16). BW, body weight; ADG, average daily gain; ADFI, average daily feed intake; G: F, ADG: ADFI. EFS, extruded full-fat soybean; ESBM, enzyme-treated soybean meal*.

### Nitrogen Digestibility

As shown in Table 4, the ESBM diet had higher AID of total nitrogen especially Histidine and Tryptophan compared to EFS diet (*P* < 0.05). However, there were no differences in AID of the other essential amino acids or the non-essential amino acid between two diets (*P* > 0.05).

**Table 4:** Effects of protein source on apparent ileal digestibility of amino acids and nitrogen of weaned pigs (%).

**Items**	**EFS**	**ESBM**	**SEM**	***P*-value**
**Essential AA (%)**				
Arginine	74.12	75.06	1.82	0.73
Histidine	83.94	88.29	1.00	0.02
Isoleucine	67.71	70.71	2.04	0.33
Leucine	64.78	69.83	2.13	0.14
Lysine	82.86	85.01	1.55	0.36
Methionine	83.38	86.46	1.39	0.16
Phenylalanine	90.09	92.07	0.62	0.06
Threonine	68.75	74.87	1.83	0.06
Tryptophan	74.41	77.64	0.90	0.04
Valine	70.09	73.75	1.62	0.15
**Non-essential AA (%)**				
Alanine	64.28	66.02	1.59	0.46
Asparagine	78.23	80.88	1.89	0.35
Cystine	75.26	75.59	1.90	0.90
Glutamine	78.98	81.92	1.89	0.31
Glycine	70.05	73.38	1.95	0.27
Proline	75.46	78.79	1.45	0.15
Serine	75.19	77.27	1.86	0.46
Tyrosine	69.34	71.92	2.67	0.52
Total nitrogen (%)	74.52	77.57	0.41	0.01

*Data were shown as the mean + SEM (n = 6). AA, amino acid. EFS, extruded full-fat soybean; ESBM, enzyme-treated soybean meal*.

### Fermentation Characteristics

As shown at Table 5, piglets in the ESBM group had higher concentrations of total SCFA, acetate and propionate than in the EFS group (*P* < 0.05). And the concentrations of total BCFA, isobutyrate and isovalerate showed a significant decrease (*P* < 0.05) in the ESBM group compared with the EFS group. As shown at Table 6, there were higher concentrations of NH_3_-N and putrescine in the EFS group than ESBM group (*P* < 0.05).

**Table 5:** Effects of protein source on volatile fatty acid composition in cecal digesta of weaned pigs (mg/g digesta).

**Items**	**EFS**	**ESBM**	**SEM**	***P*-value**
Acetate	3.23	3.96	0.31	0.04
Propionate	2.60	3.07	0.19	0.04
Butyrate	1.51	1.72	0.13	0.13
Valerate	0.68	0.67	0.08	0.89
Isobutyrate	0.28	0.22	0.02	0.01
Isovalerate	0.38	0.31	0.03	0.04
Total SCFAs^a^	8.03	9.43	0.36	<0.01
Total BCFAs^b^	0.67	0.53	0.05	0.02

a *Total SCFAs = Acetate + Propionate + Butyrate + Valerate*.

b *Total BCFAs = Isobutyrate + Isovalerate*.

*Data were shown as the mean + SEM (n = 6). SCFAs, short chain acids; BCFAs, branch chain acids. EFS, extruded full-fat soybean; ESBM, enzyme-treated soybean meal*.

**Table 6:** Effects of protein source on NH_3_-N and biogenic amine contents in cecal digesta of weaned pigs (μg /g digesta).

**Items**	**EFS**	**ESBM**	**SEM**	***P*-value**
NH_3_-N	129.33	107.16	3.79	<0.01
PUT	48.32	32.99	2.09	<0.01
CAD	28.84	24.28	1.95	0.14
SPD	14.72	13.69	1.04	0.51
SPM	2.77	2.14	0.20	0.06

*Data were shown as the mean + SEM (n = 6). PUT, putrescine; CAD, cadaverin; SPD, spermine; SPM, spermidine. EFS, extruded full-fat soybean; ESBM, enzyme-treated soybean meal*.

### Bacterial Community Richness and Biodiversity

**Figure 1:**
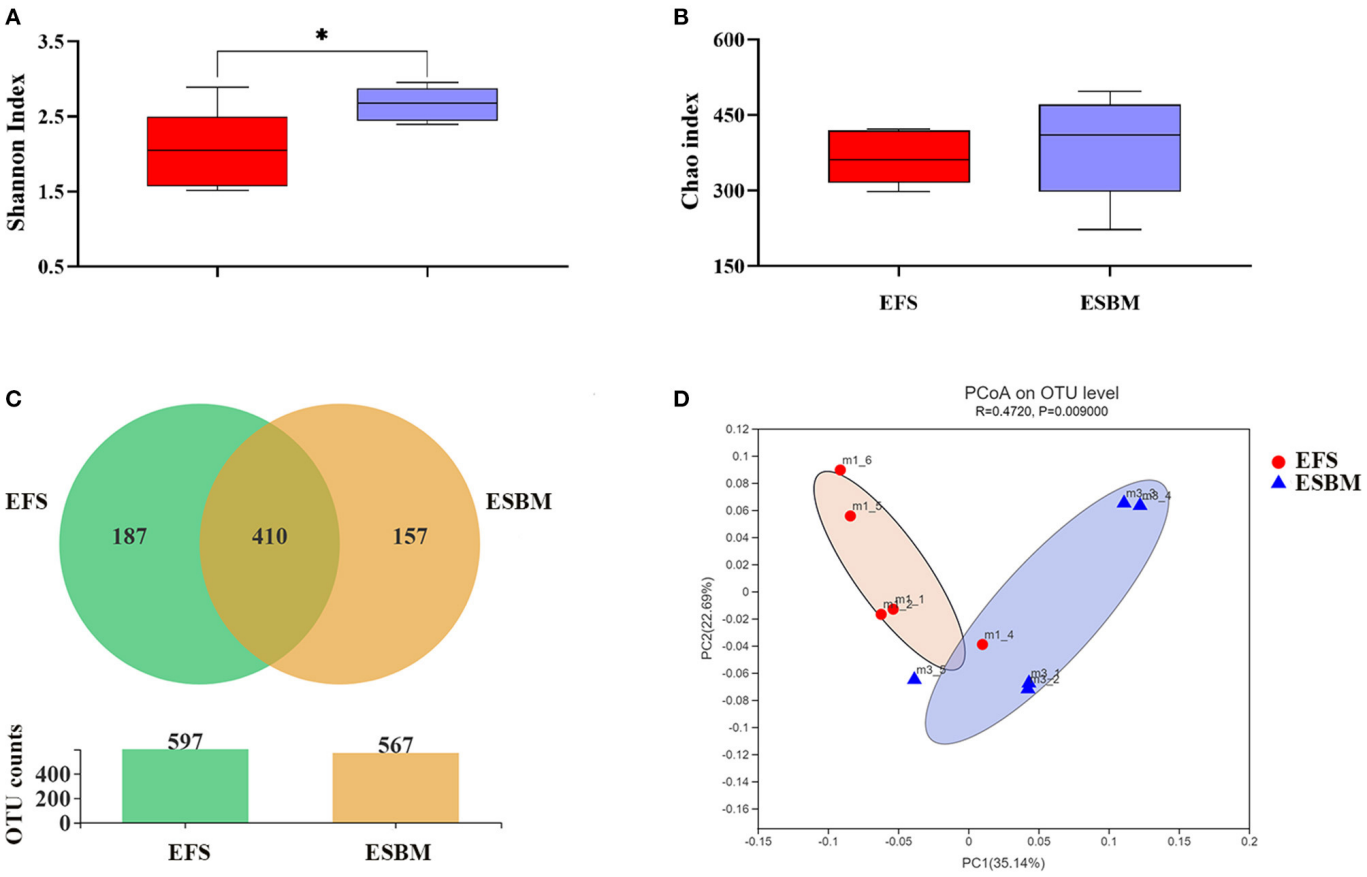
Effects of protein source on the cecal microbes at the OTU **(A)**, β-diversity **(B)** and α-diversity indexes including Shannon index **(C)** and Chao index **(D)** in piglets. The individual minipig was regarded as the experimental unit, *n* = 5 for EFS, *n* = 5 for ESBM. EFS, extruded full-fat soybean; ESBM, enzyme-treated soybean meal. *P < 0.05.

The OTUs of the cecal digesta from EFS group and ESBM group were 597 and 567, respectively, wherein 410 common OTUs have been identified (Figure 1C). The α-diversity of microbiota including Shannon index and Chao index were presented in Figures 1A,B. The ESBM diet induced higher Shannon index than the EFS (*P* < 0.05) whereas no difference was shown in Chao index between the two dietary treatments (*P* < 0.10). The β-diversity of bacterial community between EFS and ESBM was presented with PCoA, showing a different clustering of microbial communities (Figure 1D).

**Figure 2:**
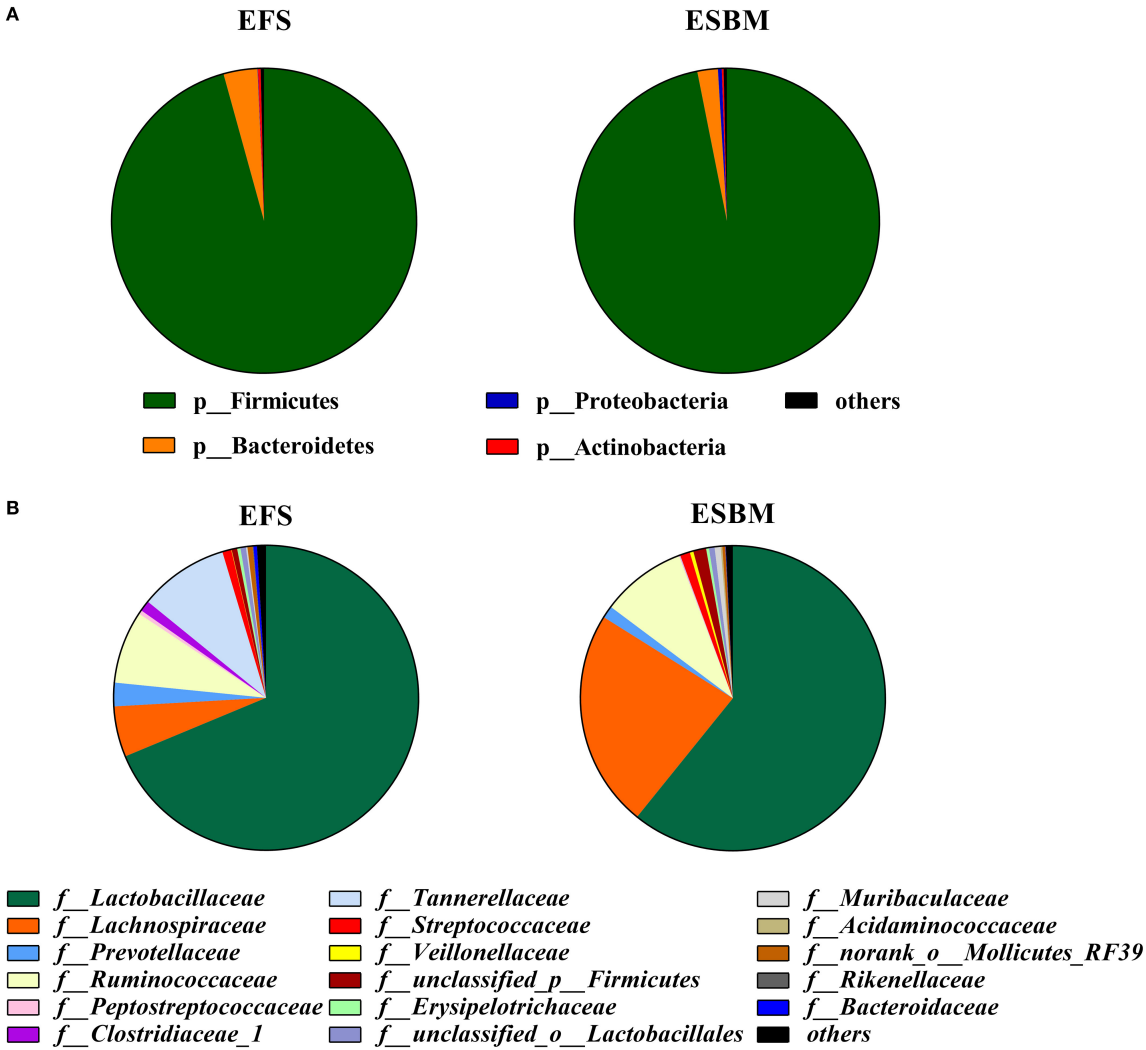
Effects of protein source on the cecal microbial composition at the phylum level **(A)** and the family level **(B)** (at least one sample relative abundance ≥0.1%) in piglets (%). The individual minipig was regarded as the experimental unit, *n* = 5 for EFS, *n* = 5 for ESBM. EFS, extruded full-fat soybean; ESBM, enzyme-treated soybean meal.

At the phylum level, Firmicutes and Bacteroidetes were the dominant bacteria and their relative abundance accounted for about 95%. However, there was no difference in microbiota at the phylum level between the two treatment groups (Figure 2A, Supplementary Table 1). A total of 17 families were detected in two dietary treatments, wherein ESBM increased the relative abundance of f_*Lachnospiraceae* and f_*unclassified_p_Firmicutes* but decreased the relative abundance of f_*Rikenellaceae* compared to the EFS diet (Figure 2B, Supplementary Table 2, *P* < 0.05).

**Figure 3:**
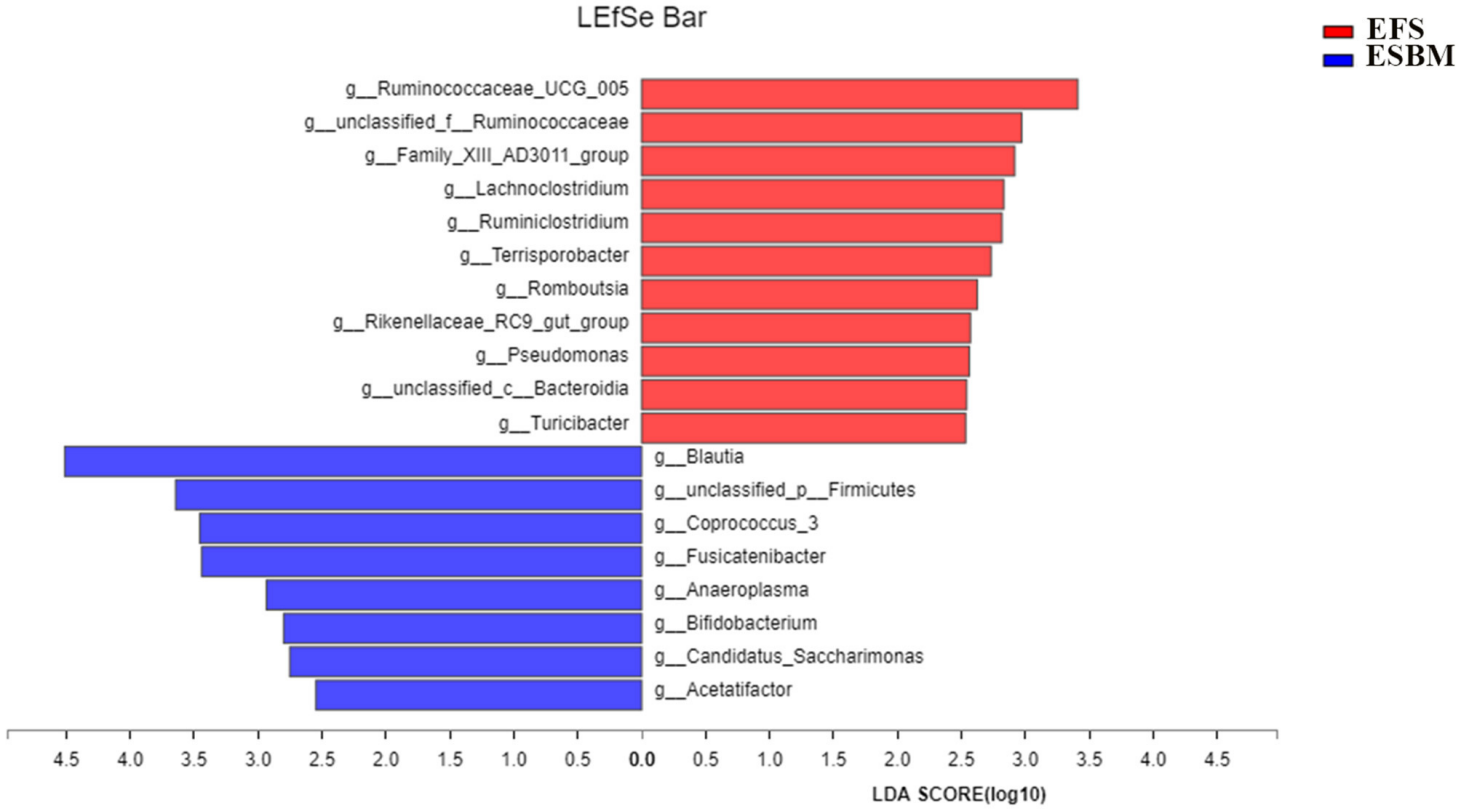
Identification of the most differentially abundant genera in cecum of the two dietary treatments. The plot was generated from Linear Discriminant Analysis Effect Size (LEfSe) analysis with CSS-normalized OTU table and displays taxa with LDA scores above 2.5 and *P*-values below 0.05. Genera enriched in the samples with EFS diet are indicated with red bars, and genera enriched in the samples with ESBM diet are indicated with blue bars. The individual minipig was regarded as the experimental unit, *n* = 5 for EFS, *n* = 5 for ESBM. EFS, extruded full-fat soybean; ESBM, enzyme-treated soybean meal.

The lefse analysis was used to identify the significantly different bacteria between the two treatment groups at the genus level. A total of 19 genera were identified to be significantly different between the two groups, including 11 genera from EFS and 8 genera from ESBM, respectively (Figure 3). The relative abundance of g_*Ruminococcaceae_UCG_005*, g_*Pseudomonas*, g_*unclassified_c_Bacteroidia* g_*Lachnoclostridium*, g_*unclassified_f_Ruminococcaceae*, g_*Romboutsia*, g_*Family_XIII_AD3011_group*, g_*Ruminiclostridium*, g_*Terrisporobacter*, g_*Rikenellaceae_RC9_gut_group*, and g_*Turicibacter* were increased in piglets fed with the EFS diet, whereas the relative abundance of g_*Blautia*, g_*unclassified_p_Firmicutes*, g_*Coprococcus_3*, g_*Fusicatenibacter*, g_*Anaeroplasma*, g_*Bifidobacterium, g_Candidatus*_*Saccharimonas*, and g_*Acetatifactor* were increased in piglets fed with the ESBM diet.

### Predicted Functional Profiles of Microbial Communities Using PICRUSt

**Figure 4:**
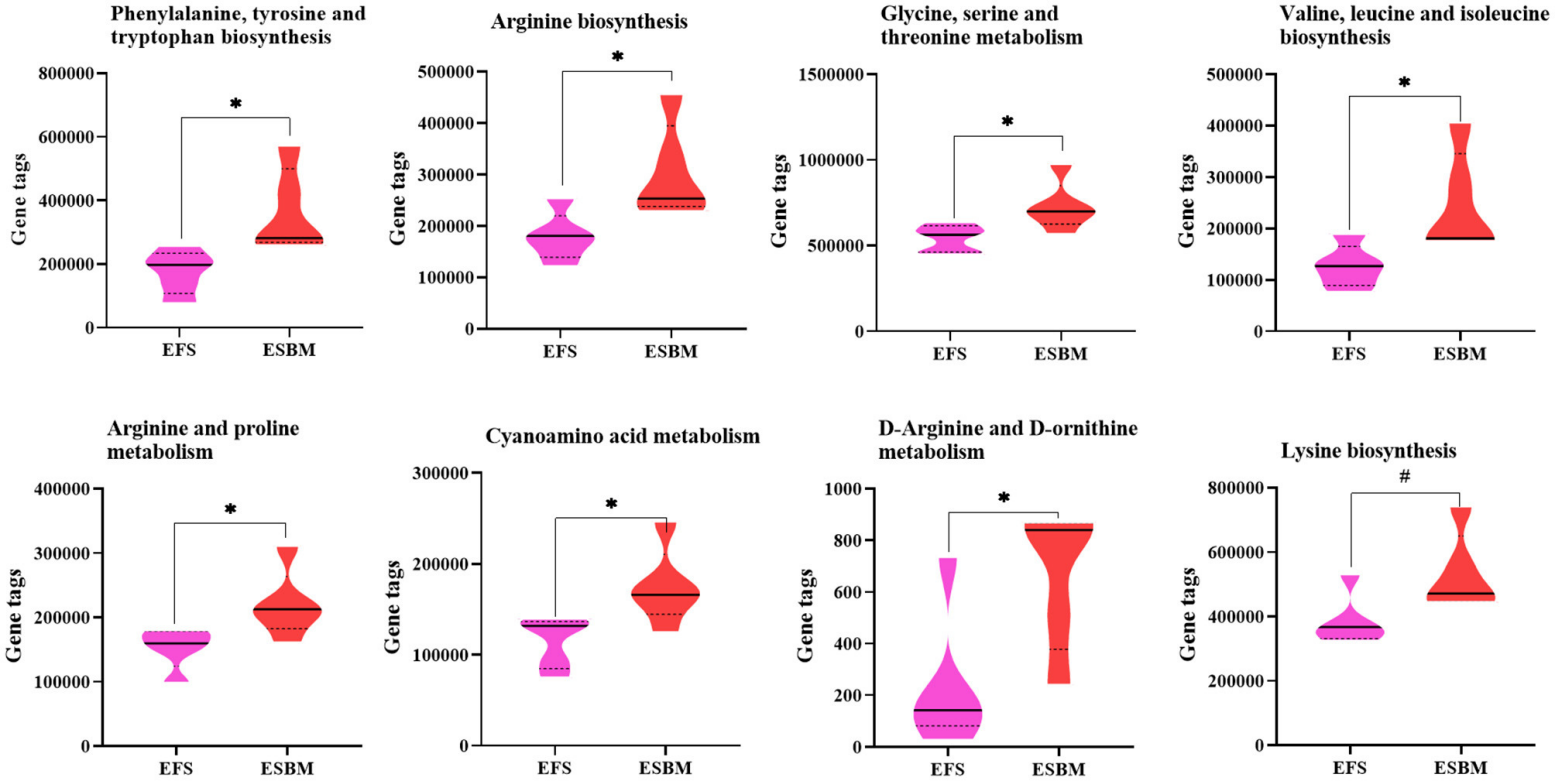
Prediction on amino acid metabolism of bacterial communities using the PICRUSt program. The individual minipig was regarded as the experimental unit, *n* = 5 for EFS, *n* = 5 for ESBM. EFS, extruded full-fat soybean; ESBM, enzyme-treated soybean meal. *P < 0.05; ^#^*P* < 0.10.

**Figure 5:**
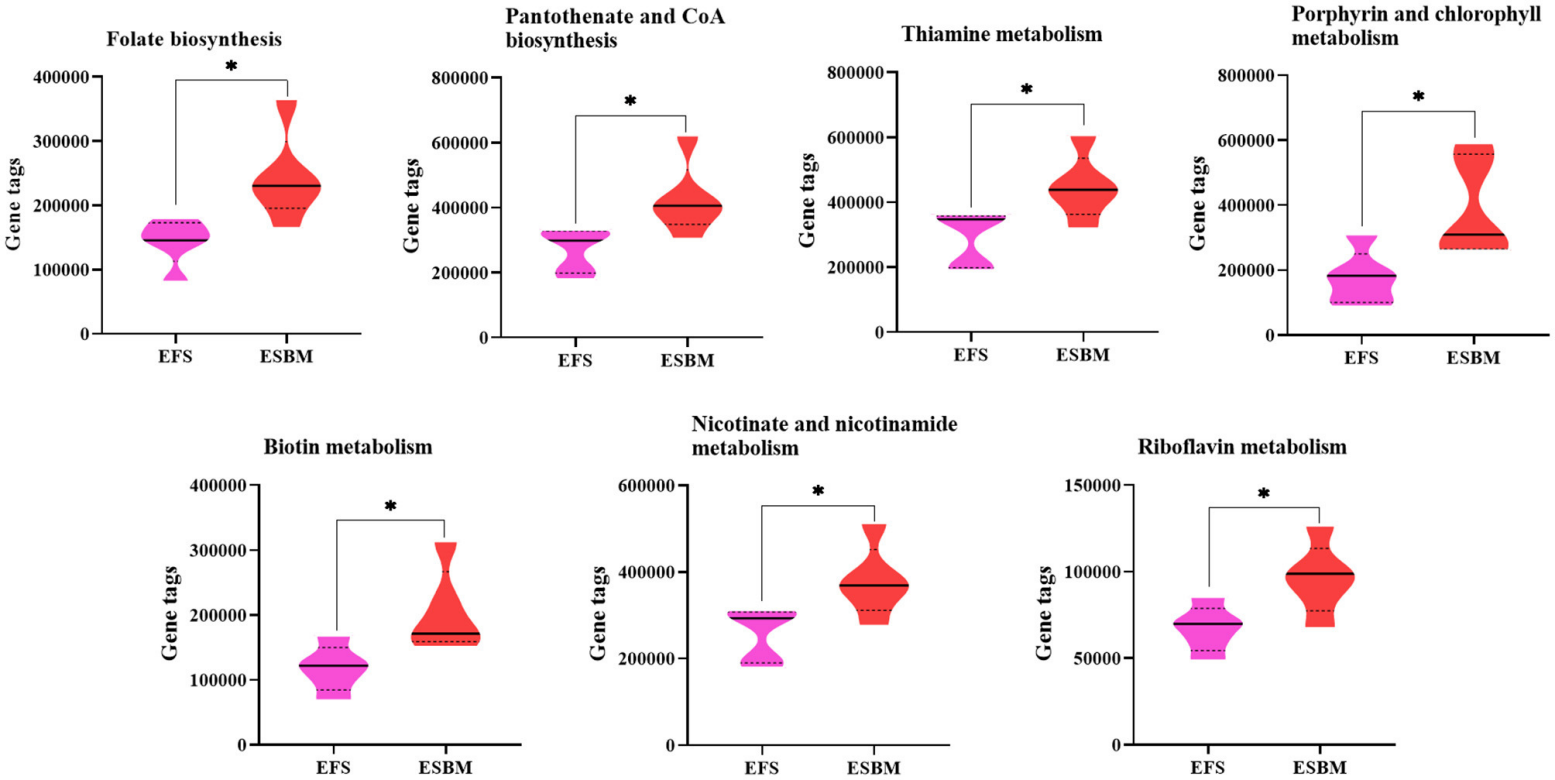
Prediction on vitamins and coenzymes metabolism of bacterial communities using the PICRUSt program. The individual minipig was regarded as the experimental unit, *n* = 5 for EFS, *n* = 5 for ESBM. EFS, extruded full-fat soybean; ESBM, enzyme-treated soybean meal. *P < 0.05.

To predict the potential function of gut bacteria on nutrient metabolism or health in piglets after feeding EFS or ESBM diets, KEGG pathways were analyzed by the PICRUSt program. A total of 285 KEGG pathways were identified and the top 50 were shown at Supplementary Figure 1. Eight pathways related to amino acid metabolism were enhanced in the ESBM group, including aromatic AA (phenylalanine, tyrosine and tryptophan) biosynthesis, branched chain AA (valine, leucine and isoleucine) biosynthesis, lysine biosynthesis, arginine biosynthesis, metabolism of glycine-serine-threonine, arginine and proline metabolism, d-arginine and d-ornithine metabolism, and cyanoamino acid metabolism (Figure 4). In the ESBM group, seven pathways related to cofactors and vitamins metabolism including folate biosynthesis, pantothenate and CoA biosynthesis, thiamine metabolism, porphyrin and chlorophyll metabolism, biotin metabolism, nicotinate and nicotinamide metabolism, and riboflavin metabolism were increased (Figure 5).

## Discussion

In the current study, the nutritional value was evaluated for soybean protein after two different processed treatments in the newly weaned piglets. Among them, the AID of crude protein in EFS and ESBM diets were 74.52 and 77.57%, respectively, which were close to the findings of previous studies (6, 9, 13). The improved crude protein digestibility of the ESBM diet could be partly explained by the lower ANFs content especially Glycinin and β-conglycinin than the EFS diet. In addition, enzyme treatment to the soybean may increase the content of small peptides, making it easy to be absorbed, which is also beneficial for improving digestibility (9). Therefore, ESBM diet significantly increased the AID of crude protein and several amino acids compared with EFS diets, which contributed to the improved the utilization of nitrogen in piglets, thereby increased the growth performance of piglets.

More importantly, the reduction in the incidence of diarrhea means that the weaning stress of piglets in the ESBM group has been better alleviated compared with the EFS group. It should be noted that reducing the protein fermentation in the hindgut can help alleviate the weaning stress of piglets (14, 15). Proteins easier to be digested tend to have better digestibility in the small intestine, which reduces the amount of protein flowing into the large intestine (16, 17). In this study, a higher AID of crude protein of the ESBM diet indicated less protein entered the hindgut and used for fermentation than the EFS diet.

Moreover, ESBM diet showed better cecal fermentation characteristics than EFS diet. The protein fermentation metabolites in the hindgut are amines, SCFAs and BCFAs wherein amines must be converted from nitrogen-containing groups, while BCFAs are only produced from the fermentation of three branched chain amino acids, leucine, isoleucine and valine (17, 18). Therefore, the concentration of amines and BCFAs could reflect the fermentation of proteins in the hindgut (18). The concentration of BCFAs, NH_3_-N and biogenic amine were lower in the ESBM group than in the EFS group, suggesting that the protein fermentation in the hindgut of the ESBM group was relatively alleviated. Notably, the ESBM group also had a higher concentration of SCFAs than the EFS group. SCFAs in the large intestine are mainly derived from the fermentation of dietary fiber, the component of plant cell wall (19). A previous study has reported that the main fermentation site of higher molecular weight insoluble non-starch polysaccharides is in the colon, while lower molecular weight anti-digestible oligosaccharides and soluble non-starch polysaccharides are fermented in the back of the small intestine and cecum (20). Enzyme treatment may damage the structure of the cell wall in a higher extent compared to extrusion, so the macromolecular non-starch polysaccharides were preliminarily degraded into more fermentable polysaccharides with lower polymerization degree, inducing increased SCFA production in pigs fed with the ESBM diet (21). This was further supported by the changes in NDF and ADF contents between ESBM and EFS.

The interaction and connection between diet, gut microbe and host have received increasing attention. As the main nitrogen source of the intestinal microbe, dietary protein has been proven to significantly change the bacterial community. Wang et al. reported that low protein diets improved hindgut health by preventing the proliferation of pathogenic bacteria (22). A better digestible protein source also reduced the risk of colitis under the same protein level (23, 24). In this experiment, f_*Lachnospiraceae* is the dominant family of bacteria in the cecum of piglets, second only to f_*Lactobacillaceae*. The relative abundance of f_*Lachnospiraceae* in the cecum of piglets was significantly increased with the ESBM diet and closely related to intestinal health (25). Moreover, g_*Blautia* belonging to f_*Lachnospiraceae* was the dominant genus second only to g_*Lactobacillus* in the cecum of piglets and significantly increased in the ESBM group, which could help clear gas in the intestinal lumen and relieve enteritis (26). Similarly, the relative abundance of g_*Fusicatenibacter* (a genus from f_*Lachnospiraceae*) in the piglet fed with the ESBM diet was also significantly higher than those fed EFS diet and has been found to be negatively related to human colitis and can relieve colitis in mice (27). Another child taxon of f_*Lachnospiraceae*, the g_*Coprococcus_3* was highly enriched in the ESBM group. Since g_*Coprococcus* is one of the common acetic acid and butyric acid producing bacteria, the higher relative abundance of g_*Coprococcus* could correspond with the change of butyrate production (28). In correspondence with the current study, Zhou et al. (14) reported that low-protein diets reduced protein flow into the hindgut and increased the relative abundance of g_*Coprococcus* in a pig model. However, g_*Lachnoclostridium* from the family *f_Lachnospiraceae* was found to increase in the EFS group, and it could be attributed to the shedding of intestinal epithelial cells (29).

Except to f_*Lachnospiraceae*, the change of other different bacteria related to intestinal health cannot be ignored. For instance, the relative abundance of *Bifidobacterium* has also increased significantly in the cecum of ESBM feeding piglets, which in most cases was recognized as beneficial for the gut health (30). Additionally, compared with the ESBM group, the EFS group had a higher relative abundance of f_*Rikenellaceae*, which was reported to positively correlate with the inflammation-mediated factors of colonic epithelial cells, including IL-6 and inflammatory chemokines in mice (31). However, it was worth mentioning that EFS also increased the relative abundance of specific beneficial bacteria such as g_*Terrisporobacter* related to immune enhancement and g_*Ruminiclostridium* related to improved intestinal barrier function, indicating that EFS also had the potentiality to improve intestinal health (32, 33). Taken together, ESBM may have higher potentiality to improve the intestinal health of piglets than EFS, as inferred from the improved microbial profile and higher protein digestibility.

Microbiota in the hindgut influence the growth and health of the host by regulating the metabolism of nutrients and energy (34). To predict the potential function of gut bacteria on nutrient metabolism or health in piglets after feeding EFS or ESBM diets, KEGG pathways were analyzed by the PICRUSt program. Our results indicated that the functional differences existed in aspects of amino acid synthesis, coenzyme and vitamin metabolism between two dietary group. Zhao et al. (19) reported that dietary fiber regulated energy metabolism of piglets by affecting butyrate producing bacteria. Similarly, PICRUSt analysis revealed the metabolic pathways such as carbohydrate and amino acid metabolism were overrepresented in all-grass-fed geese (35). Our results were similar to previous studies in that dietary protein altered the function of intestinal microbiota. However, given the fact that the functional prediction based on 16S rRNA gene sequencing does not fully reflect the functional characteristics of the bacterial community compared with the metagenome, therefore, it should be noted that the results of our functional prediction have limited reference value.

In conclusion, ESBM had higher protein ileal digestibility than EFS, which not only resulted in improved nitrogen utilization, but also inhibited protein fermentation in the hindgut of weaned piglets. Importantly, the relative abundance of beneficial bacteria (mainly f_*Lachnospiraceae*, including g_*Blautia*, g_*Fusicatenibacter*, and *Coprococcus_3*) were increased and some pathogenic bacteria (g_*Lachnoclostridium* and f_*Rikenellaceae*) was reduced in the cecum of piglets fed with ESBM diet, which may contribute to the improvement of intestinal health and attenuation of weaning stress of piglets. Therefore, our results supported the hypothesis that ESBM can be a better protein source than EFS for the application in weaned piglet diets.

## Data Availability Statement

The data presented in the study are deposited in the (NCBI SRA) repository, accession number (PRJNA720093).

## Ethics Statement

The animal study was reviewed and approved by Animal Care and Use Ethics Committee of the China Agricultural University (Beijing).

## Author Contributions

HL and XM: conceptualization, methodology, and software. ZL: literature collection. HL and JY: writing-original draft preparation. QJ, JC, and XH: writing-reviewing and editing. BT and XM: funding acquisition. All authors contributed to the article and approved the submitted version.

## Conflict of Interest

The authors declare that the research was conducted in the absence of any commercial or financial relationships that could be construed as a potential conflict of interest.
