# Supplementary material for: Enzyme-Treated Soybean Meal Replacing Extruded Full-Fat Soybean Affects Nitrogen Digestibility, Cecal Fermentation Characteristics and Bacterial Community of Newly Weaned Piglets
Source: Front Vet Sci. 2021 May 20;8:639039. doi: 10.3389/fvets.2021.639039 (PMC8173081; doi:10.3389/fvets.2021.639039)
Supplement: Supplementary file 1 [file Data_Sheet_1.docx]

**Supporting information**

**Table S1. Effects of protein source on the relative abundance of cecal microbes at the phylum level (at least one sample relative abundance ≥ 0.1%).**

|  | EFS | ESBM | SEM | *P*-value |
| --- | --- | --- | --- | --- |
| p__Firmicutes | 86.22 | 97.04 | 10.10 | 0.32 |
| p__Bacteroidetes | 12.91 | 2.06 | 9.86 | 0.30 |
| p__Tenericutes | 0.55 | 0.31 | 0.25 | 0.36 |
| p__Proteobacteria | 0.06 | 0.39 | 0.23 | 0.22 |
| others | 0.27 | 0.21 | 0.06 | 0.33 |

Data were shown as the mean + SEM (n= 5). EFS, extruded full-fat soybean; ESBM, enzyme-treated soybean meal.

**Table S2. Effects of protein source on the relative abundance of cecal microbes at the family level (at least one sample relative abundance ≥ 0.1%).**

| Bacteria, % | EFS | ESBM | SEM | *P*-value |
| --- | --- | --- | --- | --- |
| *f__Lactobacillaceae* | 68.72 | 60.82 | 9.12 | 0.41 |
| *f__Lachnospiraceae* | 5.37 | 23.11 | 6.75 | 0.03 |
| *f__Ruminococcaceae* | 7.71 | 9.02 | 2.89 | 0.67 |
| *f__Tannerellaceae* | 9.50 | 0.06 | 7.71 | 0.29 |
| *f__Prevotellaceae* | 2.50 | 1.30 | 1.71 | 0.50 |
| *f__Streptococcaceae* | 0.95 | 1.06 | 1.00 | 0.92 |
| *f__unclassified_p__Firmicutes* | 0.59 | 1.37 | 0.27 | 0.02 |
| *f__unclassified_o__Lactobacillales* | 0.58 | 0.60 | 0.56 | 0.98 |
| *f__Clostridiaceae_1* | 1.15 | 0.02 | 0.71 | 0.19 |
| *f__norank_o__Mollicutes_RF39* | 0.55 | 0.31 | 0.25 | 0.35 |
| *f__Muribaculaceae* | 0.15 | 0.63 | 0.35 | 0.24 |
| *f__Erysipelotrichaceae* | 0.35 | 0.30 | 0.12 | 0.69 |
| *f__Peptostreptococcaceae* | 0.41 | 0.05 | 0.15 | 0.07 |
| *f__T34* | 0.00 | 0.37 | 0.23 | 0.18 |
| *f__Bacteroidaceae* | 0.37 | 0.04 | 0.26 | 0.27 |
| *f__Veillonellaceae* | 0.04 | 0.38 | 0.17 | 0.11 |
| *f__Family_XIII* | 0.23 | 0.07 | 0.08 | 0.09 |
| *f__Acidaminococcaceae* | 0.01 | 0.18 | 0.11 | 0.19 |
| *f__Eggerthellaceae* | 0.13 | 0.09 | 0.04 | 0.26 |
| *f__p-251-o5* | 0.19 | 0.00 | 0.16 | 0.30 |
| *f__Coriobacteriaceae* | 0.08 | 0.03 | 0.03 | 0.19 |
| *f__Rikenellaceae* | 0.08 | 0.02 | 0.02 | 0.01 |
| *f__Atopobiaceae* | 0.03 | 0.05 | 0.03 | 0.38 |
| *f__Bacteroidales_RF16_group* | 0.07 | 0.00 | 0.05 | 0.28 |
| *f__unclassified_c__Bacteroidia* | 0.05 | 0.00 | 0.03 | 0.17 |
| *f__Enterococcaceae* | 0.03 | 0.01 | 0.02 | 0.37 |
| Others | 0.15 | 0.13 | 0.03 | 0.50 |

Data were shown as the mean + SEM (n= 5). EFS, extruded full-fat soybean; ESBM, enzyme-treated soybean meal.

**Figure S1.** Prediction on Pathway Level 3 (Top 55) of bacterial communities using the PICRUSt program. The individual minipig was regarded as the experimental unit, n = 5 for EFS, n = 5 for ESBM. EFS, extruded full-fat soybean; ESBM, enzyme-treated soybean meal.

**
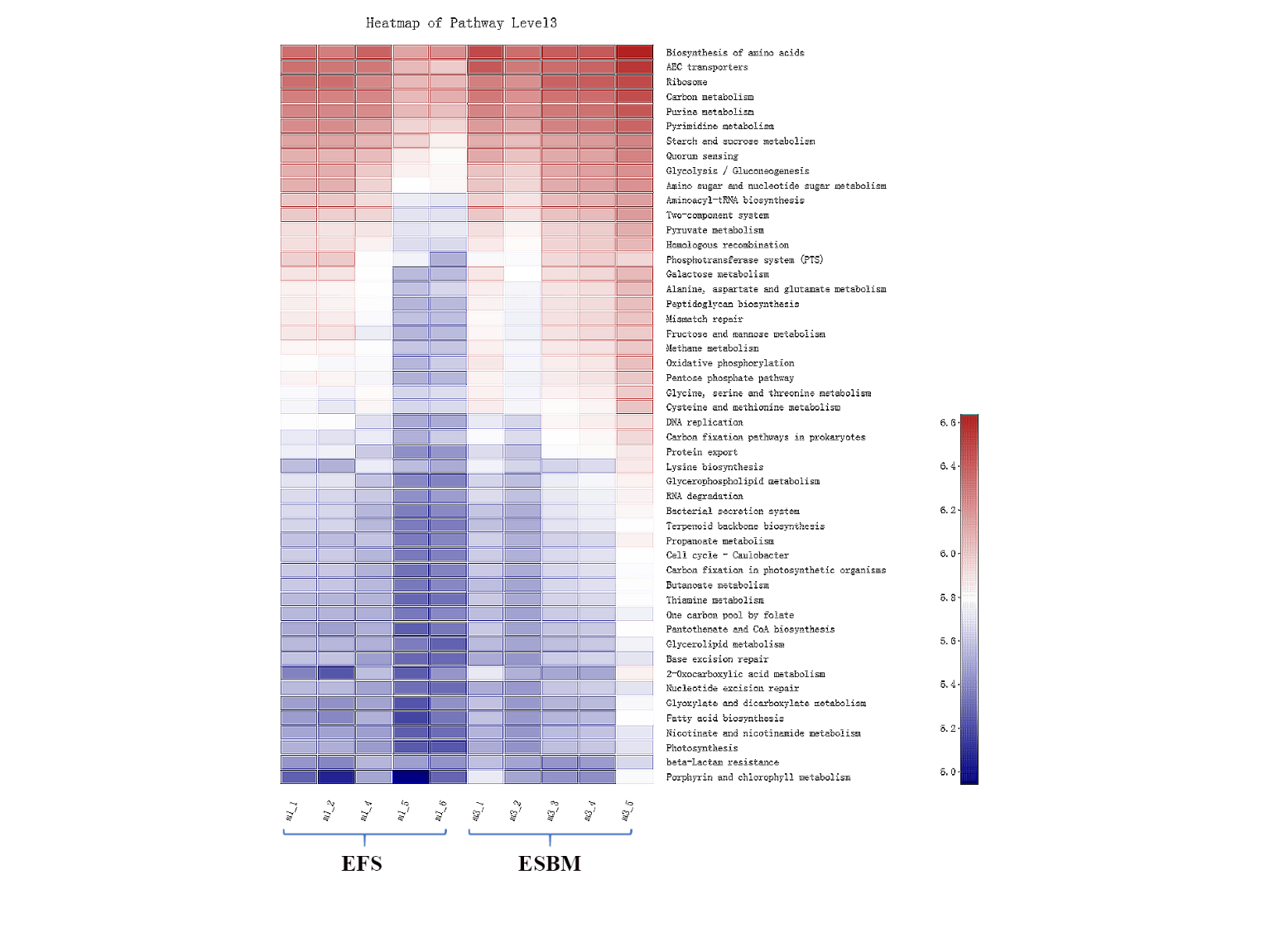
**
